# Mr. George Stranahan

**Published:** 1895-08

**Authors:** 


Mr. George Stranahan, son of Dr. C. W. Stranahan, of Erie, Pa., died July 22, 1895, from injuries received in a bicycle accident. To avert a collision with an inexperienced young woman rider, Mr. Stranahan threw himself, receiving a concussion that resulted in cerebral hemorrhage. Dr. Roswell Park was summoned together with a number of physicians in Erie, but their efforts to save life were futile. Young Mr. Stranahan had been a student at Buffalo University medical college for three years. Dr. C. W. Stranahan, his father, is one of the best known physicians in Erie and will receive the sympathy of a wide circle of professional and other friends.

